# Data Completeness and Recorded Advanced HIV Categories in a Regional Registry Extract from Northwestern Romania: A Retrospective Audit of 192 Records and Diagnosis Trends, 2013–2023

**DOI:** 10.3390/idr18050121

**Published:** 2026-10-19

**Authors:** Andrada Abrudan Secară, Timea Claudia Ghitea, Andrei Csep, Camelia Florina Iova, Eugen-Călin Secară, Ioana Ivan, Manuela Gabriela Urlan, Diana Bei, Annamaria Pallag

**Affiliations:** 1 Doctoral School of Biomedical Sciences, Faculty of Medicine and Pharmacy, University of Oradea, 410073 Oradea, Romania; abrudan.andrada@student.uoradea.ro (A.A.S.); apallag@uoradea.ro (A.P.); 2 Public Health Directorate of Bihor County, Libertății Street 34, 410042 Oradea, Romania; 3 Department of Pharmacy, Faculty of Medicine and Pharmacy, University of Oradea, 410073 Oradea, Romania; 4 Department of Psycho-Neuroscience and Recovery, Faculty of Medicine and Pharmacy, University of Oradea, 410073 Oradea, Romania; camyowa@yahoo.com; 5 Department of Psychology, Babeș-Bolyai University, Mihail Kogălniceanu 1, 400347 Cluj-Napoca, Romania; eugen.secara@ubbcluj.ro (E.-C.S.); ioana.ivan@ubbcluj.ro (I.I.); 6 Clinical Emergency County Hospital of Bihor, 410067 Oradea, Romania; urlanmanuelagabriela@gmail.com; 7 Department of Medical Disciplines, Faculty of Medicine and Pharmacy, University of Oradea, 410073 Oradea, Romania; diana.bei@didactic.uoradea.ro

**Keywords:** HIV, registry data, data completeness, surveillance, late diagnosis, advanced HIV disease, antiretroviral therapy, Romania, routinely collected health data

## Abstract

Background: Regional HIV data can support surveillance only when the meaning, timing, and completeness of core variables are explicit. We audited a regional HIV registry extract from Bihor County, Northwestern Romania, and described recorded diagnoses during 2013–2023. Methods: The supplied extract contained 192 individual records. Completeness analyses used all records. Annual-count analyses used 154 records with a diagnosis year between 2013 and 2023; 22 records diagnosed before 2013 and 16 with unknown diagnosis year were excluded from temporal analyses. A prespecified recorded advanced-category proxy was defined as CDC stage C and/or immunologic category 3 among records with interpretable A1–C3 staging. Drug indicators were recoded as non-mutually exclusive antiretroviral classes. A Poisson model quantified change in annual recorded counts; it was not interpreted as incidence because population denominators were unavailable. Results: Diagnosis year was available in 176/192 records (91.7%), residence in 149/192 (77.6%), interpretable CDC staging in 119/192 (62.0%), and at least one ART entry in 112/192 (58.3%). Exact diagnosis date, CD4 count, viral load, ART initiation date, retention in care, viral suppression, and transmission route were not represented as dedicated variables in the supplied extract. Annual recorded diagnoses ranged from 6 to 21. There was no evidence of a linear change in annual counts (incidence-rate ratio per year 0.972, 95% CI 0.925–1.022; *p* = 0.274). Among 119 staged records, 48 (40.3%) met the recorded advanced-category proxy; the timing of staging relative to diagnosis was unknown. NRTI exposure was the most frequently documented ART class, but treatment sequence and current-regimen status could not be reconstructed. Conclusions: The extract supports a data-quality and descriptive audit, not estimates of HIV incidence, confirmed late diagnosis, treatment effectiveness, or the HIV care continuum. Standardized capture of diagnosis date, CD4 count, viral load, staging date, ART initiation and current regimen, transmission route, and follow-up status is required for stronger regional surveillance.

## 1. Introduction

HIV remains a major global public-health challenge. UNAIDS estimated that 41.0 million people were living with HIV and 1.2 million people acquired HIV in 2025 [1]. In the WHO European Region, 105,922 HIV diagnoses were reported for 2024, including 24,164 in the European Union/European Economic Area (EU/EEA), with substantial variation between subregions and countries [2].

Romania has a distinctive HIV history shaped by the pediatric epidemic of the late 1980s and early 1990s and by contemporary adult transmission. National and regional publications continue to report new diagnoses and advanced disease at presentation, but comparisons across settings depend on consistent case definitions and complete clinical data [3,4].

Late HIV diagnosis has a consensus surveillance definition based on CD4 count at diagnosis and/or an AIDS-defining event, with additional rules for evidence of recent infection and previous diagnosis abroad [5]. WHO defines advanced HIV disease in adults and adolescents as a CD4 count below 200 cells/mm^3^ or WHO stage 3 or 4 disease [6]. Neither concept can be derived reliably from an undated CDC category alone. Therefore, records containing CDC stage C or category 3 may identify clinically important advanced categories, but they cannot establish late diagnosis unless timing and supporting laboratory information are available [7,8].

Routinely collected health data can reveal both epidemiological patterns and weaknesses in data capture. Transparent reporting requires clear cohort construction, variable provenance, coding rules, missing-data handling, and denominators, as emphasized by STROBE and its RECORD extension [9,10]. This is especially important when an analytic extract contains only a subset of variables that may exist elsewhere in clinical or surveillance systems.

The primary objective of this study was to quantify the availability of core demographic, diagnostic, staging, and treatment variables in a supplied regional HIV registry extract from Bihor County. Secondary objectives were to describe annual recorded diagnoses during 2013–2023, characterize recorded CDC categories, and summarize documented ART-class exposure without inferring current regimens or treatment effectiveness.

## 2. Theoretical Framework

This study is grounded in the concept that the epidemiological value of routinely collected health data depends not only on the number of available records, but also on their completeness, temporal specificity, consistency, provenance, and fitness for the intended purpose. A registry extract may be adequate for describing documented cases while remaining insufficient for estimating incidence, identifying late diagnosis, reconstructing treatment trajectories, or evaluating clinical outcomes. Accordingly, data quality is understood here as a multidimensional construct that determines which surveillance questions can be answered validly from the available information.

The framework distinguishes between three interconnected levels: data capture, indicator construction, and epidemiological interpretation. At the data-capture level, essential variables must be represented using standardized definitions and linked to clearly documented dates and sources. At the indicator-construction level, the available variables must correspond directly to the accepted definition of the outcome being measured. At the interpretation level, conclusions must remain within the boundaries imposed by the study denominator, variable completeness, temporal coverage, and data-generating process. Weakness at any of these levels may lead to misclassification or to conclusions that exceed the informational capacity of the dataset.

This distinction is particularly important in HIV surveillance. Late HIV diagnosis is defined using CD4 count and/or an AIDS-defining event at or near the time of diagnosis, subject to additional classification rules [5]. Advanced HIV disease is likewise defined using immunological or clinical criteria [6]. Therefore, an undated CDC category cannot independently establish either late diagnosis or WHO-defined advanced HIV disease. In the present study, stage C and/or immunologic category 3 is consequently treated as a recorded advanced-category proxy rather than as a validated clinical outcome. The same principle applies to antiretroviral therapy: binary drug indicators without initiation dates, discontinuation dates, or regimen sequence can describe documented exposure but cannot determine the current regimen, adherence, treatment effectiveness, or viral suppression.

The framework also draws on the principles of transparent reporting for observational studies and routinely collected health data established by STROBE and RECORD [9,10]. These principles emphasize explicit cohort construction, variable definitions, coding procedures, data provenance, missing-data assessment, and the limitations associated with secondary data use. Enhancements to established HIV surveillance systems similarly underline the importance of deduplication, linkage between clinical and laboratory sources, and continuous data-quality assessment [8].

Based on this framework, the present registry extract is evaluated according to whether its variables are fit for four intended purposes: describing the available records, examining annual recorded diagnoses, characterizing documented CDC categories, and summarizing recorded ART-class exposure. The framework does not assume that a field absent from the supplied extract is necessarily absent from the full clinical record or source registry. Instead, it supports a conservative interpretation in which each conclusion is explicitly linked to the variables, definitions, dates, and denominators available for analysis.

## 3. Materials and Methods

### 3.1. Study Design and Setting

We conducted a retrospective audit of a regional HIV registry extract from Bihor County, Northwestern Romania. The analysis used routinely collected information compiled from regional public-health, laboratory, and specialist clinical sources. Because the supplied file could not establish whether all eligible people with HIV in the county were captured, the study was treated as an audit of the available extract rather than a population-based cohort.

### 3.2. Data Source and Cohort Construction

**Figure 1:**
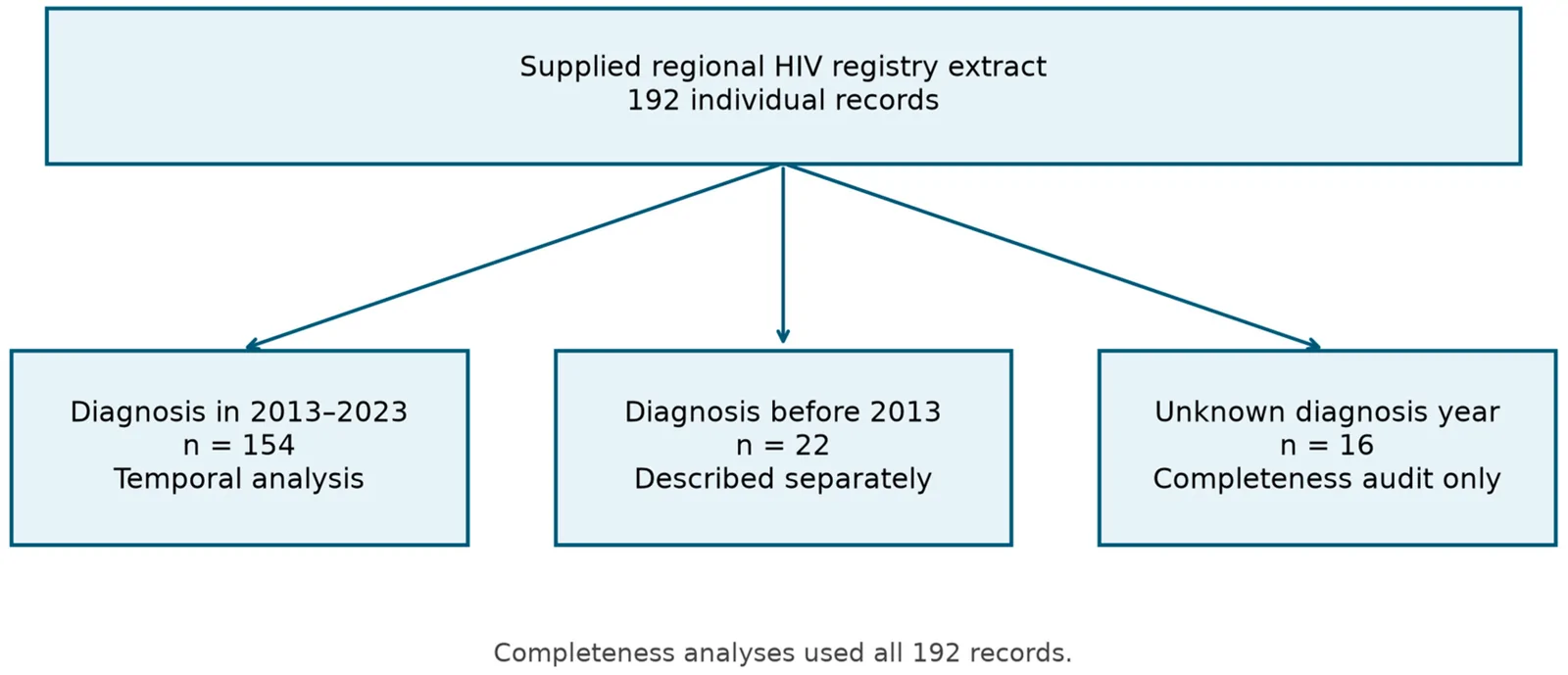
Cohort construction and analysis denominators. Temporal analyses used the 154 records with a diagnosis during 2013–2023; completeness analyses used all 192 records.

The supplied SPSS version 30 (IBM Corp., Armonk, NY, USA) file contained 192 rows, each treated as an individual record, and 39 variables. Completeness analyses included all 192 records. Diagnosis year was available for 176 records: 154 had a year between 2013 and 2023 and formed the temporal cohort, while 22 had a diagnosis before 2013. Sixteen records had no diagnosis year and were retained only in the overall completeness audit. The date on which the source systems were queried or the analytic extract was frozen was not documented in the supplied materials and should be confirmed by the authors before submission (Figure 1).

### 3.3. Variables and Data-Quality Definitions

Variables represented in the supplied extract were sex, age, CDC category, diagnosis year, residence, death year, and binary indicators for individual antiretroviral drugs or fixed-dose combinations. Diagnosis year, residence, interpretable CDC stage, and any ART entry were assessed for availability. A variable was considered unavailable when its field was blank or missing. The absence of a dedicated field in the supplied extract was described as ‘not represented in the extract’ and was not assumed to prove absence from the full clinical record or source registry.

The age field lacked a documented reference date and contained 10 zero values and one missing value. Consequently, age was not interpreted as age at diagnosis and was not used in risk-factor analyses. Sex was complete for all records. Residence was coded as urban or rural when available.

### 3.4. CDC Staging and Recorded Advanced-Category Proxy

CDC categories A1–A3, B1–B3, and C1–C3 were considered interpretable [11]. Stage N and blank fields were classified as uninterpretable. The supplied extract did not include the date of staging or reliably identify whether the category was assigned at HIV diagnosis, at entry to specialist care, or later. For descriptive purposes only, a recorded advanced-category proxy was defined as stage C and/or category 3. This proxy was not labelled late diagnosis or WHO advanced HIV disease because CD4 count, AIDS-event timing, and evidence of recent infection were unavailable [5,6].

### 3.5. ART Recoding

Drug indicators were recoded into five non-mutually exclusive classes: nucleoside/nucleotide reverse transcriptase inhibitors (NRTIs), non-nucleoside reverse transcriptase inhibitors (NNRTIs), protease inhibitors (PIs), integrase strand-transfer inhibitors (INSTIs), and pharmacokinetic boosters. Fixed-dose combinations contributed to each relevant component class. Ritonavir and cobicistat-containing products contributed to the booster indicator; ritonavir was not counted as an independent active PI when represented as a booster. Because start dates, stop dates, regimen sequence, and current-treatment status were absent, results were interpreted as documented historical or cumulative exposure.

### 3.6. Statistical Analysis

Categorical variables are reported as *n* (%). Annual recorded diagnosis counts were summarized for 2013–2023. A Poisson generalized linear model with calendar year as a continuous predictor quantified linear change in annual counts. No population offset was used because county population denominators and testing volumes were not supplied; accordingly, the resulting incidence-rate ratio describes change in recorded counts and is not an HIV incidence estimate. The analysis was performed in Python 3.11.15 (Python Software Foundation, Beaverton, OR, USA) using pandas, SciPy, statsmodels, and pyreadstat.

### 3.7. Ethics and Reporting

The study was reported in accordance with applicable STROBE and RECORD principles [9,10]. The original materials reported approval from the Research Ethics Committee of the Faculty of Medicine and Pharmacy, University of Oradea (CEFMF/2, 31 October 2024), the Bihor Public Health Directorate Ethics Committee (15644, 10 November 2023), and the Bihor County Emergency Clinical Hospital Ethics Committee (43609, 14 December 2023; 43702, 15 December 2023). The authors should verify that the final wording of the waiver and data-sharing plan is identical to the approvals.

## 4. Results

### 4.1. Cohort and Extract Completeness

The extract contained 192 records: 154 with a diagnosis during 2013–2023, 22 diagnosed before 2013, and 16 with unknown diagnosis year. Sex was recorded for all records (128 male and 64 female). A non-zero age was present in 181 records, but the age reference date was unknown; 10 records had age coded as zero and 1 was missing.

**Figure 2:**
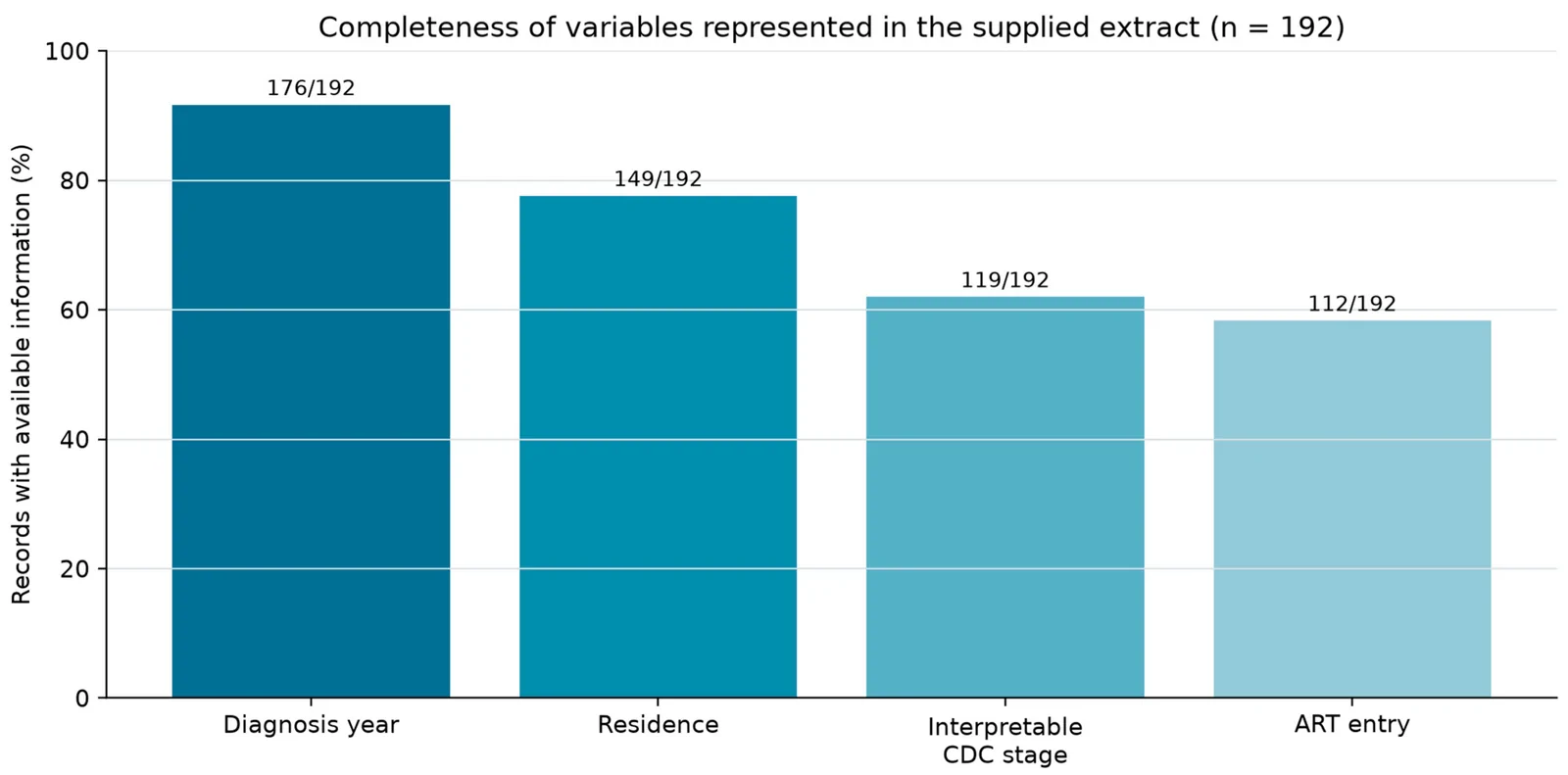
Availability of core variables represented in the supplied analytic extract. Variables absent as dedicated fields are not shown in the graph.

Diagnosis year was available in 176 (91.7%), residence in 149 (77.6%), interpretable CDC staging in 119 (62.0%), and at least one ART entry in 112 (58.3%). Missingness clustered in the 16 records with unknown diagnosis year: residence was available in 1/16, interpretable staging in 3/16, and an ART entry in 1/16 (Table 1 and Figure 2).

### 4.2. Annual Recorded Diagnoses, 2013–2023

**Figure 3:**
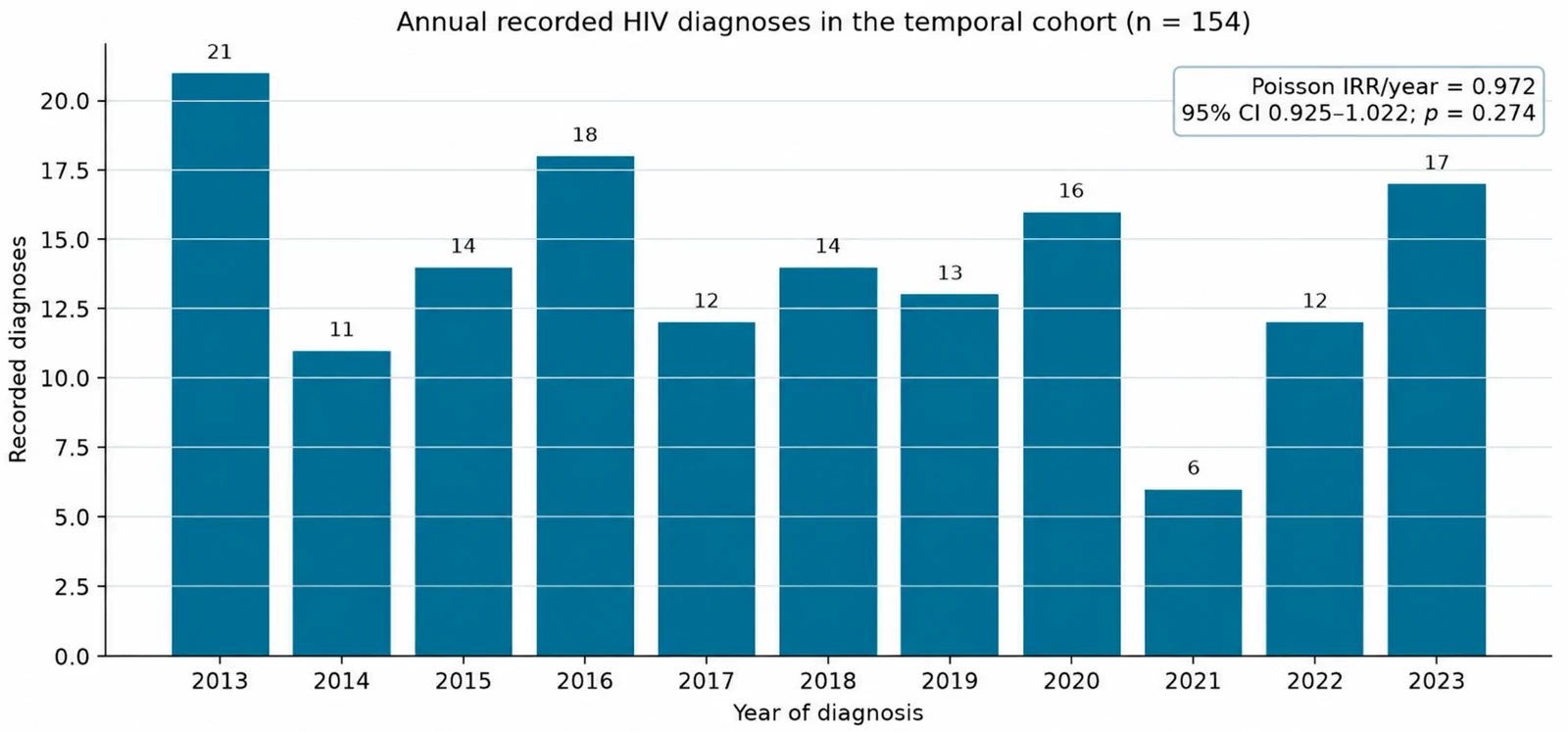
Annual recorded HIV diagnoses in the 2013–2023 temporal cohort. The Poisson estimate quantifies change in recorded counts without a population offset and must not be interpreted as incidence.

Annual recorded diagnoses ranged from 6 to 21, with a median of 14 and a mean of 14.0. The Poisson model did not show evidence of a linear change in counts (IRR per year 0.972, 95% CI 0.925–1.022; *p* = 0.274). The model describes recorded diagnoses only and does not account for changes in testing, reporting, migration, or population size (Table 2 and Figure 3).

### 4.3. CDC Categories and Recorded Advanced-Category Proxy

An interpretable A1–C3 category was present in 119/192 records (62.0%). The remaining 73 records comprised 31 stage-N entries and 42 blank entries. Among interpretable records, stage A was recorded in 48, stage B in 37, and stage C in 34. The recorded advanced-category proxy was present in 48/119 staged records (40.3%). In the 2013–2023 temporal cohort, it was present in 41/105 staged records (39.0%) (Table 3).

### 4.4. Documented ART-Class Exposure

At least one drug or fixed-dose combination was recorded in 112/192 records (58.3%). NRTI exposure was most common, followed by boosters, PIs, INSTIs, and NNRTIs. Class indicators were non-mutually exclusive. Two records with an ART entry did not include a mapped NRTI, which is compatible with incomplete or historical documentation but does not identify an active regimen (Table 4).

## 5. Discussion

This audit showed that the supplied regional HIV extract can support a transparent description of record availability, recorded diagnoses by year, CDC-category distribution, and cumulative ART-class documentation. It cannot support population incidence, confirmed late diagnosis, treatment effectiveness, or care-continuum outcomes. Reframing the study around these supported objectives is the principal methodological correction.

The absence of a statistically detectable linear change in annual recorded counts should not be interpreted as epidemiological stability. Recorded counts may vary with testing volume, referral patterns, reporting completeness, mobility, and disruptions such as the COVID-19 pandemic. European surveillance data demonstrate substantial subregional heterogeneity, while Romanian studies have reported a considerable burden of advanced disease among newly diagnosed patients [2,3,4]. Direct comparison with the present extract is limited because this study lacks CD4 count, viral load, transmission category, and a confirmed population denominator.

The recorded advanced-category proxy was common among staged records, but its clinical meaning is limited by uncertain timing. The updated consensus definition of late HIV diagnosis relies on CD4 count and AIDS-defining events at diagnosis, with reclassification for recent infection and exclusion of previous diagnoses abroad [5]. WHO advanced HIV disease similarly requires a CD4 count below 200 cells/mm^3^ or WHO stage 3 or 4 disease [6]. A CDC category recorded at an unknown time can flag records for review but cannot substitute for either definition.

Missingness was not evenly distributed: records without a diagnosis year were also rarely documented for residence, staging, or ART. This pattern may reflect heterogeneous source records, historical carry-over, or incomplete linkage, but the supplied data cannot distinguish these mechanisms. A source-to-variable mapping and validation sample against the original records would be needed before attributing missing extract fields to failure of the underlying registry.

ART-class counts also require conservative interpretation. Contemporary European guidelines emphasize complete regimen and monitoring information [12]. In the present extract, brand names, generic names, individual agents, and combination products were represented as binary indicators without dates. These fields can describe documented exposure but cannot reconstruct simultaneous regimens, switches, or response.

The year-to-year pattern merits cautious contextual interpretation. The lowest recorded count occurred in 2021, followed by higher counts in 2022 and 2023. This sequence is compatible with delayed testing, diagnosis, referral, or data entry during the COVID-19 period, followed by partial recovery of services; however, the present extract contains no testing-volume, service-access, or reporting-delay variables with which to test that explanation. International evidence confirms that HIV testing and related services were disrupted during the pandemic, but it does not establish that the same mechanism caused the local fluctuation [13,14]. The absence of a significant linear trend also does not exclude non-linear change or short-term disruption, particularly with only 11 annual observations and relatively small counts [14,15].

The findings should also be distinguished from Romanian clinical cohorts that specifically enrolled newly diagnosed patients and had access to CD4 counts, viral load, symptoms, or AIDS-defining conditions [3,4]. Such studies can estimate the proportion presenting late or with advanced disease within their defined clinical populations, whereas the present analysis evaluates the informational capacity of a heterogeneous registry extract. The observed 40.3% advanced-category proxy among interpretable staged records may indicate a subgroup requiring clinical review, but comparison of this percentage with late-diagnosis estimates would conflate different definitions, denominators, and time points [5,16,17].

This distinction has practical consequences for regional public-health decision making. Counts alone cannot reveal whether apparent changes reflect transmission, testing activity, case ascertainment, migration, transfer of care, or delayed reporting. Similarly, a binary indication that a drug was ever recorded cannot establish whether a person is currently receiving ART or has achieved viral suppression. Using such fields as performance indicators could therefore misdirect resources or overstate program outcomes. Until dated laboratory and care-engagement variables are available, the defensible uses of the extract are case reconciliation, completeness monitoring, identification of records requiring source verification, and descriptive reporting with explicit denominators [18,19,20,21].

The clustering of missing fields among records without a diagnosis year further suggests that completeness should be monitored jointly rather than variable by variable. A record-level completeness score, stratified by source system and calendar period, could help identify whether deficits arise predominantly in older records, specific reporting pathways, or failed linkage. Enhancements to other national HIV surveillance systems have similarly emphasized deduplication, linkage, data quality, and the incorporation of laboratory and care information [8]. For the regional system, periodic validation against a sample of source records would be essential before interpreting improvements in extract completeness as improvements in the underlying registry [22,23,24,25].

### 5.1. Minimum Data Set for Future Regional Surveillance

A prospective regional data model should distinguish the date and source of each observation and should support late-diagnosis, linkage, treatment, retention, and suppression indicators. The following minimum fields would materially improve interpretability (Table 5) [26,27,28,29].

### 5.2. Strengths and Limitations

Strengths include the inclusion of all 192 supplied individual records, explicit denominator separation, reproducible recoding of ART classes, and conservative interpretation of staging. Limitations are substantial: the extract was not verified as a complete population registry; the extraction date and source-to-variable mapping were unavailable; age had no documented reference date; CDC staging lacked a date; laboratory, transmission, and longitudinal care variables were absent; ART indicators lacked timing; and small-area sensitive data create disclosure risks. Missingness may be informative rather than random, and no source-record validation was available to quantify misclassification or distinguish absent source data from extraction loss. The annual-count model was based on only 11 observations, assumed a log-linear mean structure, and lacked population and testing-volume offsets; it therefore had limited capacity to characterize non-linear or service-related changes. These limitations prevent causal, predictive, treatment-effectiveness, and care-continuum analyses.

## 6. Conclusions

The supplied regional HIV registry extract contains useful descriptive information but has insufficient clinical and temporal detail for several commonly reported HIV surveillance outcomes. Among 192 records, diagnosis year, residence, interpretable CDC category, and an ART entry were available in 91.7%, 77.6%, 62.0%, and 58.3%, respectively. Annual recorded diagnoses during 2013–2023 showed no statistically detectable linear change, but the analysis did not estimate incidence. Forty-eight of 119 staged records contained stage C and/or category 3; this should be treated as an undated recorded advanced-category proxy, not confirmed late diagnosis. Future surveillance should prioritize dated CD4, viral load, staging, ART, and care-engagement fields together with explicit data provenance.

## Figures and Tables

**Table 1:** Availability, completeness, and interpretability of core demographic, clinical, and treatment variables in the regional HIV registry extract (*N* = 192).

Variable	Eligible *n*	Available	Interpretation
Sex	192	192 (100.0%)	M/F field complete
Age	192	181 (94.3%) non-zero	Reference date unknown; 10 zero values; 1 missing
Diagnosis year	192	176 (91.7%)	154 in 2013–2023; 22 before 2013
Residence	192	149 (77.6%)	Urban/rural when recorded
Interpretable CDC category	192	119 (62.0%)	A1–C3; N/blank treated as uninterpretable
Any ART entry	192	112 (58.3%)	Historical/current status not distinguishable
Exact diagnosis date	192	Not represented	No dedicated variable in supplied extract
CD4 count/viral load	192	Not represented	Late diagnosis and viral suppression not derivable
ART start/stop dates	192	Not represented	Current regimen and time to treatment not derivable
Transmission route/retention	192	Not represented	Care-continuum analyses not derivable

**Table 2:** Annual distribution of recorded HIV diagnoses in the temporal cohort, 2013–2023 (*n* = 154).

Year	*n*	% of Temporal Cohort
2013	21	13.6%
2014	11	7.1%
2015	14	9.1%
2016	18	11.7%
2017	12	7.8%
2018	14	9.1%
2019	13	8.4%
2020	16	10.4%
2021	6	3.9%
2022	12	7.8%
2023	17	11.0%

**Table 3:** Distribution of recorded CDC clinical–immunological categories and the advanced-category proxy in the regional HIV registry extract (*N* = 192).

Recorded Category	*n*	% of All 192	% of 119 Staged
A1	20	10.4%	16.8%
A2	26	13.5%	21.8%
A3	2	1.0%	1.7%
B1	12	6.2%	10.1%
B2	13	6.8%	10.9%
B3	12	6.2%	10.1%
C1	0	0.0%	0.0%
C2	3	1.6%	2.5%
C3	31	16.1%	26.1%
N	31	16.1%	—
(blank)	42	21.9%	—
Stage C and/or category 3	48	25.0%	40.3%

Because the date and clinical context of staging were unavailable, these percentages cannot be reported as the prevalence of late HIV diagnosis or WHO-defined advanced HIV disease. Percentages among staged records were calculated using the 119 records with an interpretable A1–C3 category. Stage N and blank entries were considered uninterpretable. The advanced-category proxy was defined as stage C and/or immunological category 3.

**Table 4:** Documented antiretroviral drug-class exposure in the regional HIV registry extract (*N* = 192).

Class	*n*	% of All 192	% of 112 ART-Documented
NRTI	110	57.3%	98.2%
Booster	64	33.3%	57.1%
PI	63	32.8%	56.2%
INSTI	57	29.7%	50.9%
NNRTI	29	15.1%	25.9%

The drug fields do not support claims about guideline concordance, adherence, treatment response, switching, resistance, or viral suppression because regimen dates and laboratory outcomes are absent. ART-class indicators were non-mutually exclusive; therefore, percentages do not sum to 100%. The data represent documented historical or cumulative exposure and should not be interpreted as current treatment regimens.

**Table 5:** Proposed minimum data set for comprehensive regional HIV surveillance and registry-based evaluation.

Domain	Core Fields	Primary Use
Case identity	Stable pseudonymous identifier; source system	Deduplication and linkage
Diagnosis	Exact date; first-ever vs. previously diagnosed; last negative test	Incident diagnosis and late-diagnosis classification
Transmission	Probable transmission category; country of birth/previous diagnosis where permitted	Stratified surveillance
Immunology	CD4 count and date at/near diagnosis	Late diagnosis and advanced disease
Virology	Viral load and date at baseline and follow-up	Treatment response and viral suppression
Clinical stage	Classification system, category, date, and defining event	Valid temporal interpretation
Treatment	ART start/stop dates; complete regimen; reason for switch	Time to ART and current regimen
Care engagement	Visit dates; transfer; death; loss to follow-up	Retention and outcome ascertainment
Data provenance	Extraction date; variable dictionary; validation status	Reproducibility and auditability

The proposed fields are intended to support reliable case linkage, classification of late diagnosis and advanced HIV disease, monitoring of ART initiation and current treatment, assessment of retention and viral suppression, and reproducible data-quality auditing.

## Data Availability

All data is publicly available at https://osf.io/eqn82 (accessed on 20 August 2026). The anonymized data supporting the findings of this study are publicly available in the Open Science Framework repository at the address provided by the authors.

## Abbreviations

The following abbreviations are used in this manuscript:

AIDS	Acquired immunodeficiency syndrome
ART	Antiretroviral therapy
CDC	Centers for Disease Control and Prevention
CD4	Cluster of differentiation 4
EACS	European AIDS Clinical Society
ECDC	European Centre for Disease Prevention and Control
EU/EEA	European Union/European Economic Area
GDPR	General Data Protection Regulation
HIV	Human immunodeficiency virus
INSTI	Integrase strand transfer inhibitor
NNRTI	Non-nucleoside reverse transcriptase inhibitor
NRTI	Nucleoside/nucleotide reverse transcriptase inhibitor
PI	Protease inhibitor
UNAIDS	Joint United Nations Programme on HIV/AIDS
WHO	World Health Organization
